# RNA methylation in mammalian development and cancer

**DOI:** 10.1007/s10565-021-09627-8

**Published:** 2021-07-17

**Authors:** Peizhe Song, Subiding Tayier, Zhihe Cai, Guifang Jia

**Affiliations:** Synthetic and Functional Biomolecules Center, Beijing National Laboratory for Molecular Sciences, Key Laboratory of Bioorganic Chemistry and Molecular Engineering of Ministry of Education, College of Chemistry and Molecular Engineering, Peking University, Beijing, 100871 China

**Keywords:** *N*^6^-methyladenosine, 5-methylcytosine, *N*^1^-methyladenosine, RNA metabolism, Stem cell fate determination, Embryonic development, Cancer progression

## Abstract

Similar to epigenetic DNA and histone modifications, epitranscriptomic modifications (RNA modifications) have emerged as crucial regulators in temporal and spatial gene expression during eukaryotic development. To date, over 170 diverse types of chemical modifications have been identified upon RNA nucleobases. Some of these post-synthesized modifications can be reversibly installed, removed, and decoded by their specific cellular components and play critical roles in different biological processes. Accordingly, dysregulation of RNA modification effectors is tightly orchestrated with developmental processes. Here, we particularly focus on three well-studied RNA modifications, including *N*^6^-methyladenosine (m^6^A), 5-methylcytosine (m^5^C), and *N*^1^-methyladenosine (m^1^A), and summarize recent knowledge of underlying mechanisms and critical roles of these RNA modifications in stem cell fate determination, embryonic development, and cancer progression, providing a better understanding of the whole association between epitranscriptomic regulation and mammalian development.

## Introduction

Overall, gene expression is tightly orchestrated with mammalian developmental processes such as stem cell fate determination, embryonic development, and cancer susceptibility; and gene expression programs are precisely modulated with mRNA metabolism and protein synthesis during development. Recently, RNA chemical modifications upon diverse RNA subtypes have emerged as a regulatory mechanism to coordinate cellular transcriptomes and proteomes in different physiological processes. To date, in addition to the canonical four RNA residues, over 170 different types of post-synthesized modifications have been identified in cellular RNA (Boccaletto et al. 2018). Ribosomal RNA (rRNA) and transfer RNA (tRNA), the highly abundant RNA species, are heavily modified. With the development of RNA modification detection methods, internal RNA modifications have been characterized increasingly in message RNA (mRNA) and non-coding RNA (ncRNA) (Dominissini 2014; Li et al. 2016b). Of these, *N*^6^-methyladenosine (m^6^A) serves as the best characterized RNA modification, and occurs in mRNA (Dominissini et al. 2012), rRNA (Maden 1986), and ncRNA (Patil et al. 2016). Other internal RNA modifications such as 5-methylcytosine (m^5^C) and *N*^1^-methyladenosine (m^1^A) are embedded into coding and non-coding RNAs (Squires et al. 2012; Delatte et al. 2016; Dominissini et al. 2016; Li et al. 2016a). Similar with DNA epigenetics and histone modifications, RNA modifications can be dynamically deposited, removed, and recognized by their specific cellular components (known as "writers", "erasers", and "readers", respectively), which play crucial roles in modulating RNA metabolism during mammalian development and cancer (Frye et al. 2018).

**Fig. 1:**
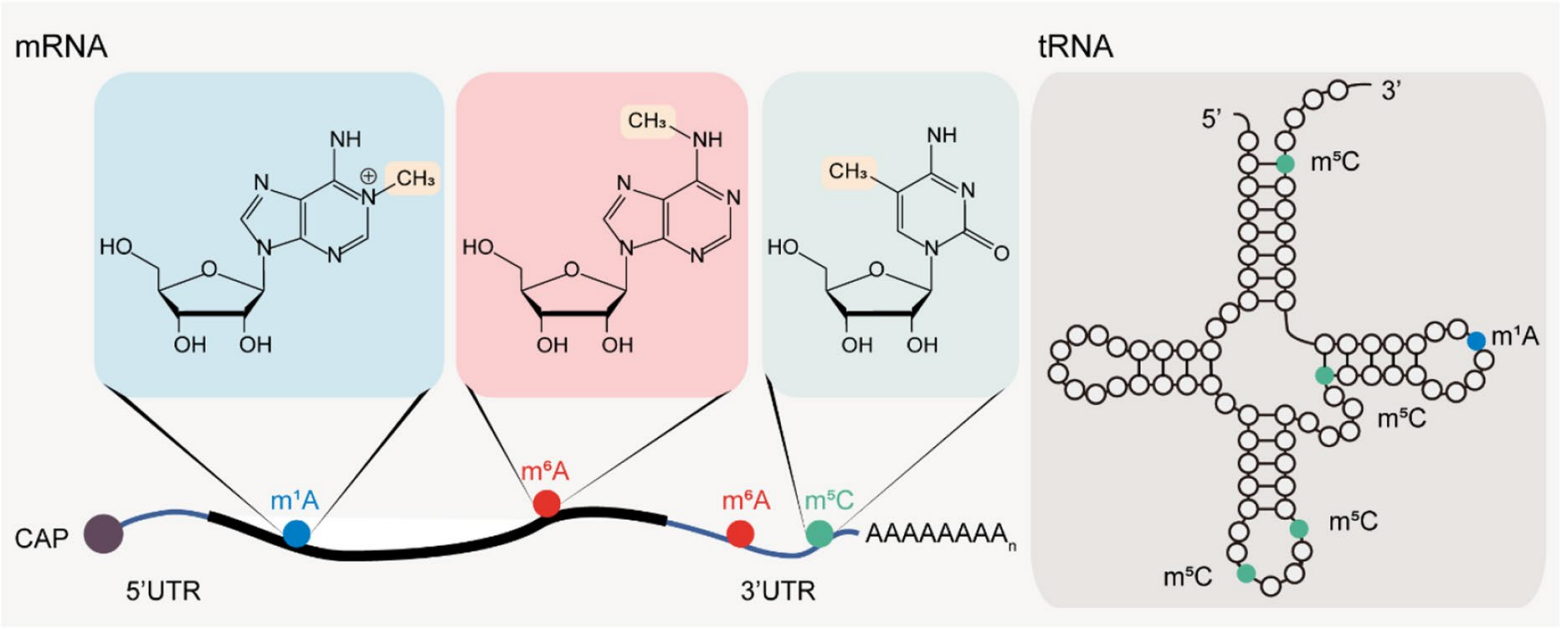
Internal RNA methylation modifications. Schematic representation of three internal RNA methylation modifications (m^1^A, m^6^A, and m^5^C) in mRNA (left panel) and tRNA (right panel). Methylated nucleotides are highly enriched in the regions where they are most frequent in mRNA and tRNA. The chemical structures and the highlighted methyl groups of these methylated nucleotides are represented in the figure

In this review, we will focus on the well-studied epitranscriptomic modifications including m^6^A, m^5^C, and m^1^A as notable examples (Fig. 1) and describe its functional mechanisms in the regulation of gene expression. Additionally, we summarize recent studies regarding the critical roles of these RNA modifications in stem cell differentiation, embryonic development, and cancer progression.

## The regulatory networks of the three well-studied RNA methylation

### *N*^6^-Methyladenosine

**Fig. 2:**
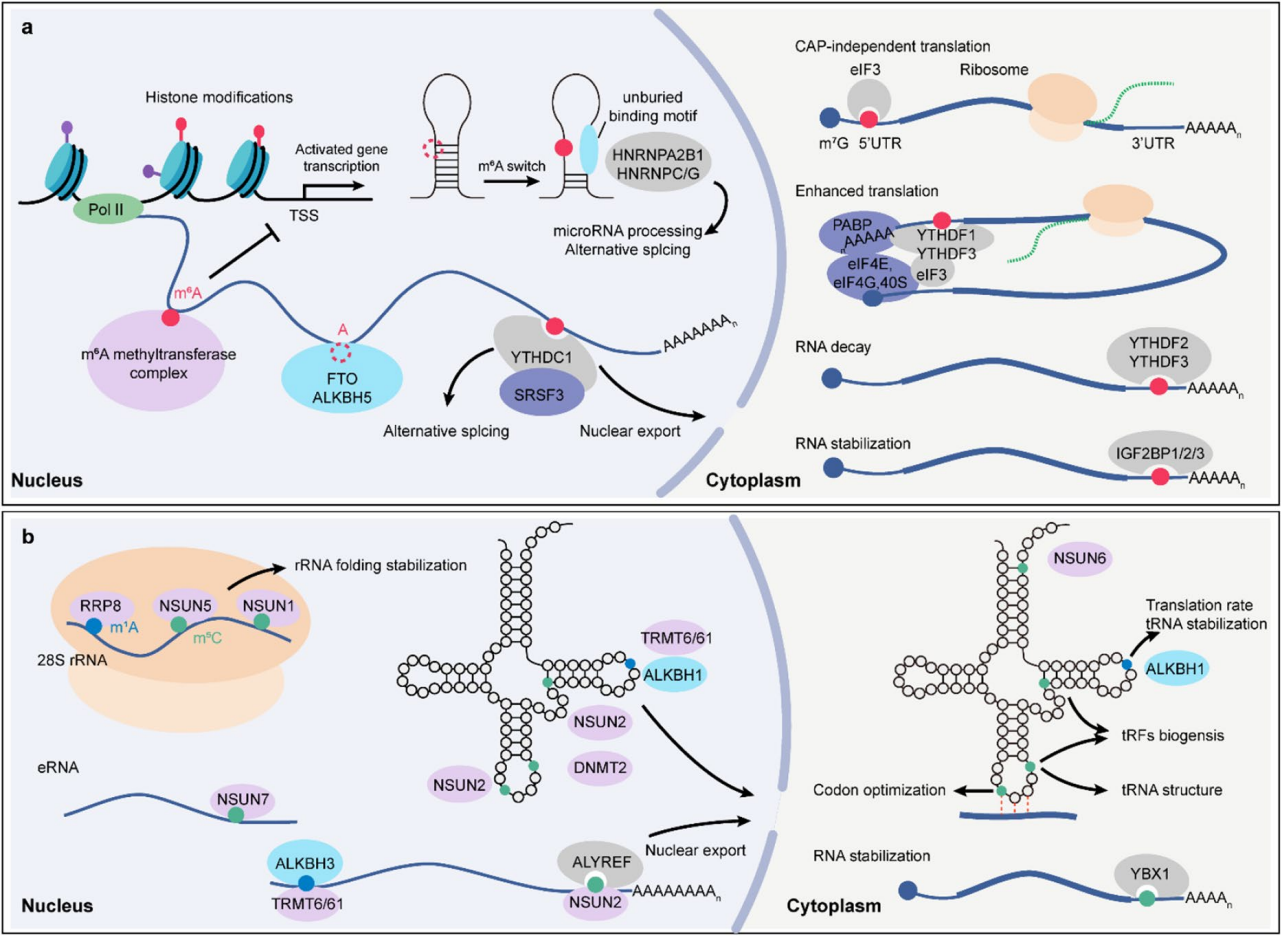
Regulation of RNA methylation in gene expression. **a** m^6^A is co-transcriptionally installed by an m^6^A methyltransferase complex and removed by demethylases FTO or ALKBH5. m^6^A binding proteins specifically recognize m^6^A-modified transcripts and function diverse roles in RNA metabolism. **b** The deposition, removal, recognition, and downstream gene expression regulation of m^5^C and m^1^A upon diverse RNA subtypes are shown

As the best characterized and the most prevalent mRNA modification (Jia et al. 2013), m^6^A occurs 0.1–0.4% in total adenosine residues (Wei et al. 1975), and mediates almost every aspect in mRNA metabolism. m^6^A greatly enriches near stop codons, 3′UTR, and long internal exons, and appears to be confined within a conserved motif DRACH (where D = A, G or U; R = A or G; H = A, C or U) (Dominissini et al. 2012; Meyer et al. 2012). m^6^A deposition within RNA polymerase II–transcribed transcripts is co-transcriptionally formed by the m^6^A writer complex comprising the asymmetric METTL3–METTL14 heterodimer, where METTL14 functions as a scaffold along with the catalytic component METTL3 to perform the methylation reaction (Wang et al. 2016a, b). The subunit of the m^6^A writer complex, WTAP, physically interacts with METTL3/14, and functions in the nuclear speckle localization of METTL3 and METTL14 (Ping et al. 2014). WTAP is required for the methylation capability, and its depletion paradoxically shows larger effects on m^6^A than does depletion of METTL3 or METTL14 (Schwartz et al. 2014; Liu et al. 2014). Furthermore, VIRMA, ZC3H13, RBM15 and/or RBM15B, and CBLL1 proteins have been characterized as the subunits of the m^6^A-methylation complex and function critical roles in m^6^A deposition (Fig. 2a). Removal of *VIRMA* results in a substantial loss of m^6^A and induces longer 3′ UTR selection (Schwartz et al. 2014; Yue et al. 2018); ZC3H13 is required for nuclear localization of the writer proteins, and its depletion leads to a reduction of m^6^A (Wen et al. 2018); RBM15 and RBM15B are RNA-binding proteins and can associate with METTL3 and promote particular methylation in mRNAs (Patil et al. 2016) or long non-coding RNA such as *XIST* (Patil et al. 2016); CBLL1 is also known as HAKAI, and its deletion causes a partial loss of m^6^A level (Fujita et al. 2002; Ruzicka et al. 2017). Besides, the formation of m^6^A is also catalyzed by METTL16, which could alone deposit m^6^A in U6 small nuclear RNA (snRNA) and U6-m^6^A-consensus sequence in the *MAT2A* transcript (Pendleton et al. 2017).

The m^6^A-conserved motif DRACH appears heavily frequent in mRNA, suggesting that there are many putative m^6^A sites in each transcript. However, only very limited DRACH sequences have been methylated. Thus, the transcript- and site-specific m^6^A deposition requires more precise regulatory mechanism. Co-transcriptional m^6^A deposition confers specificity in transcripts through the interplay of the methyltransferase complex with histone modification, H3K36me3, or transcription factors that guide it to specific promoters (Huang et al. 2019; Bertero et al. 2018). Alternatively, the selectivity of m^6^A particular locations within transcripts depends on the interaction of its writer complex with RNA polymerase II and RNA-binding proteins, such as RBM15 and RBM15B (Slobodin et al. 2017; Patil et al. 2016). Until now, details of achieving the intracellular transcript- and site-specific methylation are still not clear, and there are apparently other regulatory pathways awaiting exploration.

Unlike m^6^A methyltransferase that needs multiple complex proteins to achieve the activity, m^6^A demethylase alone removes m^6^A modification. Until now, only two Fe(II)/α-KG-dependent m^6^A demethylases, FTO and ALKBH5, have been characterized in mammals (Fig. 2a) (Jia et al. 2011; Zheng et al. 2013). FTO is the first determined m^6^A demethylase and can dynamically remove m^6^A from nuclear mRNA in vitro and in vivo, implying the reversible regulation of post-transcriptional modification upon mRNA (Jia et al. 2011). ALKBH5 is the second endogenous m^6^A demethylase and influences mRNA export in nuclear speckles (Zheng et al. 2013). Distinct from the substrate specificity of ALKBH5, FTO has been found to demethylate multiple RNA modifications. Mauer and colleagues showed that FTO has extensively higher catalytic capability for demethylating *N*^6^,2′-*O*-dimethyladenosine (m^6^A_m_), adjacent to the m^7^G cap on mRNA (termed cap m^6^A_m_), than for m^6^A (Mauer et al. 2017). Cap m^6^A_m_ was suggested to impair DCP2-mediated mRNA decapping for stabilization of mRNA (Mauer et al. 2017). Controversially, the identification of cap m^6^A_m_ writer PCIF1 reveals that cap m^6^A_m_ functions in protein translation, not in mRNA stability (Sendinc et al. 2019). In vitro biochemistry and cell-based assays showed that FTO not only can demethylate both internal m^6^A and cap m^6^A_m_ in mRNA, but also can demethylate internal m^6^A_m_ in snRNA and m^1^A in tRNA (Mauer et al. 2019; Wei et al. 2018). The crystal complex structure of human FTO bound to *N*^6^-methyldeoxyadenosine-modified ssDNA is solved and explains the catalytic mechanism how FTO recognizes and demethylates these diverse substrates (Zhang et al. 2019), revealing that m^6^A is the most favorable nucleobase substrate of FTO. Moreover, it is also found that FTO exhibits the same demethylation activity toward m^6^A and m^6^A_m_ of the same RNA sequence. Due to the total amount of m^6^A was substantially tenfold more than that of cap m^6^A_m_ in mRNAs, internal m^6^A modification is still the most favorable substrate of FTO (Wei et al. 2018; Zhang et al. 2019).

The precise regulation of m^6^A on RNA metabolism is achieved by the recognition of its binding proteins directly and indirectly. The YT521-B homology (YTH) domain was discovered by the in vitro pull-down assay with the m^6^A-modified probe (Dominissini et al. 2012). Subsequent crystal structure studies revealed that the m^6^A-dependent binding capability of YTH domain is achieved chiefly through an "aromatic pocket", in which two or three tryptophans enclose m^6^A spatially (Xu et al. 2014). The YTH domain, around 150 amino acids, is highly conserved in a wide range of eukaryotes, including human, fruit fly, yeast, and plant (Stoilov et al. 2002)*.* In mammals, there are five YTH proteins (YTHDF1-3, YTHDC1-2) tightly bound to m^6^A-modified transcripts in the cytoplasm (YTHDF1-3, YTHDC2) and nucleus (YTHDC1-2) (Wang et al. 2014a, 2015; Shi et al. 2017; Xiao et al. 2016; Roundtree et al. 2017; Wojtas et al. 2017). Additionally, other putative m^6^A readers (IGF2BP1-3, eIF3) are also identified and directly interact with the methylated transcripts through its RNA binding domain (Huang et al. 2018; Meyer et al. 2015). HNRNP family members HNRNPA2B1, HNRNPC, and HNRNPG are indirect m^6^A binding proteins and recognize the m^6^A switch, of which m^6^A unfolds the RNA–protein structures and renders transcripts more accessible, instead of m^6^A itself (Liu et al. 2015, 2017; Alarcón et al. 2015).

As the subcellular localization is known to affect the types of regulatory pathways, the separate subcellular location of m^6^A readers confer distinctive functions in gene expression, ranged from epigenetic silencing, alternative splicing, nuclear export, transcription, and chromatin state in the nucleus to RNA stability and translation efficiency in the cytoplasm (Fig. 2a). In the nucleus, YTHDC1, together with splicing factor SRSF3 but antagonize with SRSF10, recognizes exon m^6^A and promotes exon inclusion type pre-mRNA alternative splicing (Xiao et al. 2016). YTHDC1 also tunes the nuclear export of m^6^A-modified transcripts via the interaction with SRSF3 and NXF1 (Roundtree et al. 2017). Ribonucleoproteins, HNRNPC and HNRNPG, depend on the recognition of m^6^A switch to affect the occurrence of splicing events (Liu et al. 2015; Zhou et al. 2019). In addition to the mRNA regulation, HNRNPA2B1 binds to m^6^A-modified primary microRNA (pri-miRNA) transcripts and associates with the microprocessor protein DGCR8, thereby facilitating the pri-miRNA processing (Alarcón et al. 2015). The long noncoding RNA (lncRNA) XIST is highly methylated, and m^6^A promotes *XIST*-mediated gene repression by recruiting YTHDC1 (Patil et al. 2016). Markedly, recent works in mouse embryonic stem cells identified that the co-transcriptional m^6^A methylation is tightly orchestrated with chromatin state: removal of either *METTL3* or *YTHDC1* activates transcription and enhances chromatin accessibility via deposition and recognition of methylated chromosome-associated regulatory RNAs (carRNAs) (Liu et al. 2020); Li and colleagues exhibited a genome-wide correlation between METTL3-mediated methylation and chromatin modification H3K9me2 (Li et al. 2020); dysregulated m^6^A methylation by *METTL3* depletion impairs the deposition of multiple heterochromatin modifications onto intracisternal A particle (IAP) transcripts and upregulates its transcription (Xu et al. 2021); one more recent study also reported that knockout of *YTHDC1* activates these repressed retrotransposons transcripts (such as IAPs, ERVK, and LINE1) in a SETDB1-mediated H3K9me3-dependent manner. These results reveal critical roles for m^6^A in the regulation of chromatin modification, heterochromatin integrity, and retrotransposon repression (Liu et al. 2021). In the cytoplasm, YTHDF family proteins function in m^6^A-containing mature RNA metabolism. YTHDF1 mostly interacts with methylated sites near the 3′ end of transcripts and promotes the cap-dependent translation initiation and efficiency, probably via a looping association with eIF3 (Wang et al. 2015). YTHDF2 destabilizes the m^6^A-modified transcripts by recruiting CCR4-NOT deadenylation complex, which deadenylase the poly(A) tail and accelerate the degradation of targeted transcripts (Wang et al. 2014a; Du et al. 2016). YTHDF3 binds either YTHDF1 or YTHDF2 and facilitates translation and decay of m^6^A-containing transcripts, separately (Shi et al. 2017). Apart from canonical cap-dependent translation initiation, elF3 could bind 5′UTR m^6^A and initiate translation with recruitment of the 43S ribosomal complex in a cap-independent translation manner (Meyer et al. 2015). Contrary to YTHDF2 functions, IGF2BPs could enhance the stability of its targeted transcripts in an m^6^A-dependent manner, suggesting that YTHDF2 and IGF2BPs may confer specific recognition toward m^6^A-methylated sites or transcripts (Huang et al. 2018). These distinctive regulatory mechanisms of m^6^A binding proteins could basically tune every aspect of RNA processing and metabolism, reflecting the accuracy and complexity of m^6^A regulation.

### 5-Methylcytosine

Methylated cytosine residues at the position 5 are very common in DNA. Despite being discovered for decades (Dubin and Taylor 1975), m^5^C in RNA did not gain enough attention due to the lesser abundance. Until recently, with the development of high-throughput sequencing approaches, m^5^C is identified to distribute widely in various RNA subtypes, including tRNA, rRNA, mRNA, and ncRNA as well as enhance RNA (eRNA), and performs diverse functions (Bohnsack et al. 2019). In mRNA, m^5^C appears 0.4% in total cytosine residues (Squires et al. 2012), and greatly distributes either in locations near to the Argonaute-binding regions within the 3′ UTR (Squires et al. 2012) or in the vicinity of the translational start site of mRNA (Yang et al. 2017a).

Two different types of enzyme proteins have been identified as m^5^C writers, including NSUN1-7 and DNMT2 (Bohnsack et al. 2019). Members in NSUN family have respective RNA targets and catalytic mechanisms (Fig. 2b). As for rRNAs and tRNAs, NSUN1/Nop2 and NSUN5/Rcm1 are in charge of m^5^C installation in human eukaryotic 28S rRNA/yeast 25S rRNA (Sharma et al. 2013b; Schosserer et al. 2015). And these methylated rRNAs are considered to possibly function in stabilizing rRNA folding in ribosome by promoting base stack and enhancing hydrogen bond with guanine (Hayrapetyan et al. 2009; Motorin and Helm 2010). Meanwhile, *Rcm1*-deleted yeast leads to a structural change in rRNA and facilitates read-through of the premature stop codon under oxidative stress (Schosserer et al. 2015). NSUN2, NSUN6, and DNMT2 are responsible for the methylation of tRNA (Tuorto et al. 2012; Haag et al. 2015; Goll et al. 2006). NSUN2 mediates the m^5^C methylation within the anticodon (C34) of pre-tRNA^Leu(CAA)^ (Brzezicha et al. 2006), likely contributing to the translation process by affecting codon-anticodon interaction. However, those m^5^C located out of anticodon are thought to function in regulating secondary structure of tRNA, such as DNMT2-mediated methylation at position 38 (Tuorto et al. 2012). Besides, it is spotted that the dysregulated NSUN2- and DNMT2-mediated m^5^C is related to the formation of tRNA fragments (tRFs) from tRNA cleavage, further causing a mistranslation process upon cellular and environmental cues (Blanco et al. 2016). Moreover, NSUN3 and NSUN4 are synthesized on cytoplasmic ribosomes and localized in mitochondria, enabling m^5^C deposition in mitochondrial tRNA and 12S rRNA, respectively (Nakano et al. 2016; Metodiev et al. 2014). NUSN7 is thought to be related to eRNA methylation and functions in metabolic stress response (Aguilo et al. 2016). In addition to rRNAs and tRNAs, NSUN2 also acts as the m^5^C writer in mRNAs and promotes m^5^C-mediated mRNA export (Yang et al. 2017a). As the traceable hm^5^C is found in RNAs from those species lacking of TET genes (Fu et al. 2014), m^5^C can be oxidized by the TET-family enzymes. TET2 functions in promoting the conversion of m^5^C to hm^5^C on tRNA and regulating the processing or stability of different classes of tRFs (He et al. 2021). Meanwhile, TET2-mediated oxidation of m^5^C in tRNA leads to a significant increase in translation in vitro, but not obvious in vivo (Shen et al. 2020). Additionally, TET proteins in *Drosophila* have been revealed to catalyze the oxidative hydroxylation of m^5^C to hm^5^C in mRNA, where hm^5^C is notably present in coding sequences and can favor mRNA translation (Delatte et al. 2016). In mammalian mRNA, TET2 mediates oxidation of m^5^C and its disruption causes the transcriptome-wide upregulation of m^5^C, such as the ones in the 3′UTR of *SOCS3*, further influencing the pathogen infection–induced myelopoiesis (Shen et al. 2018).

Functionally, ALYREF is identified as m^5^C reader and promotes nuclear export of m^5^C-containing transcripts (Yang et al. 2017a). Using pull-down along with LC–MS assay, YBX1 is captured as m^5^C binding protein, and coordinately maintains mRNA stability in zebrafish with Pabpc1a, thereby enhancing the stabilization of m^5^C-containing transcripts (Yang et al. 2019) (Fig. 2b).

### *N*^1^-Methyladenosine

As another important RNA modification, m^1^A is methylated at the *N*^1^ position of adenosine and appears in tRNA, rRNA, mRNA, and mitochondrial (mt) transcripts (El Yacoubi et al. 2012; Sharma et al. 2013a; Li et al. 2017a; Safra et al. 2017). The m^1^A methylation endows various RNA types with a positive charge that may affect its structures and interactions with potential partner proteins. m^1^A is first detected in yeast tRNA^Phe^ (RajBhandary et al. 1966) and presents at the position 9, 14, 58 of tRNA (Anderson and Droogmans 2005). Among them, m^1^A 58 upon tRNA is highly conserved in bacteria, archaea, and eukaryote, and functions in tRNA stabilization, especially that of eukaryotic initiator tRNA^iMet^ (Anderson et al. 1998; Liu et al. 2016). Contrary to the high abundance of m^1^A in tRNA, m^1^A occurs in 0.015–0.054% of total cytosine residues in mammalian mRNA (Dominissini et al. 2016; Li et al. 2016a). The development of m^1^A sequencing and m^1^A single-base resolution methods unravels specific m^1^A sites in mRNA and mt-encoded transcripts (Dominissini et al. 2016; Li et al. 2016a, 2017a; Safra et al. 2017). Li and colleagues identified over 400 m^1^A sites in mRNA and lncRNA, most of which are highly located within 5′ UTR (Li et al. 2017a). Additionally, m^1^A modification is prevalent in mt-encoded transcripts and interferes with mitochondrial translation (Li et al. 2017a; Safra et al. 2017).

TRMT6 and TRMT61 are tRNA m^1^A writers (Vilardo et al. 2020; Guy and Phizicky 2014), while RRP8 installs m^1^A in 28S rRNA (Peifer et al. 2013). Human mt-tRNAs have been reported to contain m^1^A sites at position 9 and 58, which could be methylated by TRMT10C and TRMT61B, respectively (Vilardo et al. 2012; Chujo and Suzuki 2012). As for mRNAs, TRMT6 and TMRT61 complex also functions in the regulation of m^1^A methylation in very few mRNAs (Safra et al. 2017; Li et al. 2017a). TRMT10C deposits m^1^A in the mitochondrial ND5 mRNA (Safra et al. 2017). TRMT61B also acts as a mitochondrial m^1^A methyltransferase, and TRMT61B-mediated m^1^A deposition in mt-mRNAs interferes with translation (Li et al. 2017a). ALKBH1 and ALKBH3 mediate the m^1^A demethylation process, in which ALKBH1 demethylates m^1^A 58 in tRNA and plays critical roles in attenuating translation elongation (Liu et al. 2016; Chen et al. 2019b); and ALKBH3 is established as the only known m^1^A eraser in mRNA (Woo and Chambers 2019; Li et al. 2016a) (Fig. 2b). Still now, there are no specific m^1^A binding proteins identified. Therefore, more knowledge toward m^1^A readers and biological functions needs further study.

## RNA methylation functions in stem cell fate

Stem cells have been widely utilized to decipher the regulatory elements underlying cell fate decisions. The determination of cell fate is tightly orchestrated with global gene expression alterations, some of which are regulated by the epigenetic mechanism. Apart from epigenetic marks (DNA methylations and histone modifications), epitranscriptomic modifications (m^6^A and m^5^C) are also required for stem cell state maintenance, differentiation, and development.

### m^6^A functions in embryonic stem cell pluripotency

Embryonic stem cells (ESCs) reside in the early embryo blastocyst and have the pluripotent capability to form all tissues of the embryo. ESCs are notably separated into two states: naïve-state ESCs and primed epiblast stem cells (EpiSCs). Naïve-state ESCs are originated from the inner cell mass of developing pre-implantation blastocysts and represented as a naïve state, whereas EpiSCs are isolated from the post-implantation epiblasts and primed to differentiation.

**Fig. 3:**
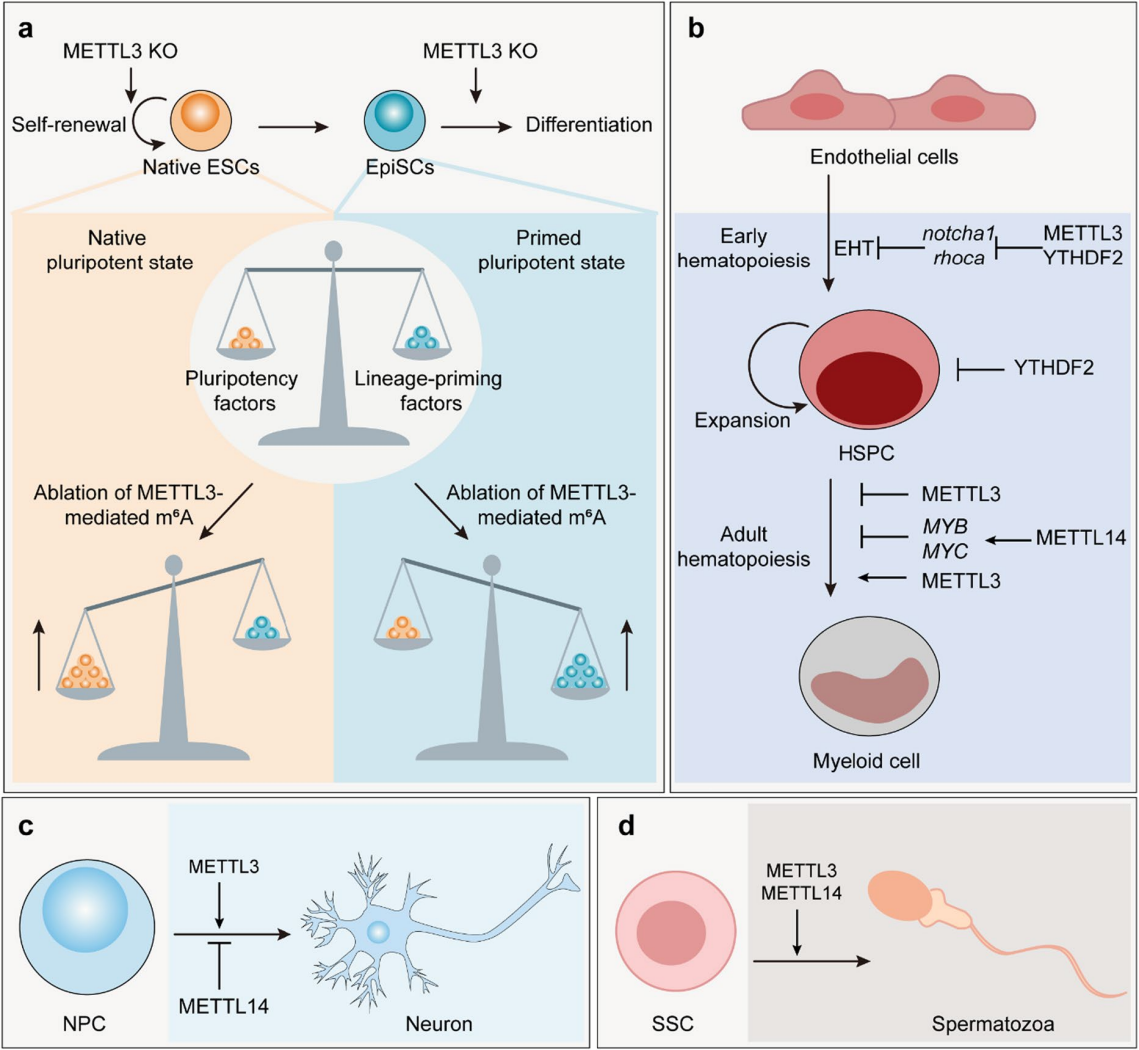
m^6^A regulation of stem cell fate determination. **a** m^6^A balances pluripotency and lineage commitment in ESCs. As m^6^A enhances the degradation of methylated transcripts, deletion of METTL3-mediated m^6^A deposition in naïve ESCs upregulates dominating pluripotency factors to maintain a hyper-naïve state. However, deficiency of METTL3 in EpiSCs boosts the already-abundant lineage-priming genes to trigger cell specification. **b** In normal hematopoiesis, m^6^A promotes the early hematopoiesis and exerts divergent effects (activation or inhibition) to myeloid differentiation in different study systems. **c** In neurogenesis, m^6^A plays conflicting roles (activation or inhibition) in NPC differentiation. **d** In spermatogenesis, m^6^A promotes the SSCs specification. ESC, embryonic stem cell; EpiSC, primed epiblast stem cell; EHT, endothelial-to-hematopoietic transition; HSPC, hematopoietic stem/progenitor cell; NPC, neural progenitor cell; SSC, spermatogonial stem cell

The transcriptome-wide m^6^A profiling in mouse and human ESCs revealed that most transcripts encoding the core pluripotency factors were methylated, implicating a possible role in affecting ESC fate determination (Batista et al. 2014). Two individual studies showed that the removal of *METTL3* in ESCs delays turnover of self-renewal state and impedes cell specification in vitro (Batista et al. 2014; Geula et al. 2015). The homozygous *METTL3-*deficient mice are embryonic lethal. Lacking *METTL3* embryos appear normal before implantation, but initiate to die at the post-implantation stage, in which *METTL*3-depleted ESCs constantly transcribe pluripotency factor *NANOG*, but fail to undertake differentiation into downstream lineages (Geula et al. 2015). Consistently, depletion of *METTL14* in embryos blocks differentiation and displays embryonic growth retardation from embryonic d 6.5, leading to embryonic lethality (Meng et al. 2019). However, other studies reported that deficiency of *METTL3* and *METTL4* in mESCs lost its self-renewal capability and stabilizes the methylated developmental regulators via the HuR/microRNA pathway (Wang et al. 2014b). These conflicting results could be explained by the association between the current stem cell state and m^6^A-mediated regulation. At the naïve pluripotent state, *METTL3-*deleted ESCs delay turnover of dominating pluripotency factors, and are locked in a hyper-naїve state. In the primed pluripotent state, deficiency of *METTL3* upregulates already-abundant lineage-commitment genes in a YTHDF2-dependent manner, diminishing self-renewal and triggering cell specification (Fig. 3a). Notably, upon Activin–NODAL signaling pathway, transcription factors Smad2/3 recruit the METTL3-METTL14-WTAP complex to facilitate m^6^A deposition and destabilize its transcriptional targets, particularly pluripotency gene *NANOG*, thereby enabling timely exit from pluripotency and differentiation into downstream lineages (Bertero et al. 2018).

Recently, increasing studies highlight the critical function of METTL3-mediated methylation in the regulation of chromatin state upon mammalian ESCs. It is reported that dysregulated m^6^A methylation in *METTL3*-knockout mESCs enhances carRNA levels and activates chromatin state and downstream transcription (Liu et al. 2020). The carRNAs include promoter-associated RNAs, eRNAs, and repeat RNAs. The METTL3-mediated m^6^A modifications on carRNAs (such as repeat RNA LINE1, elements of the long interspersed element-1 family) are recognized by YTHDC1 and processed to decay through the nuclear exosome targeting-mediated nuclear degradation pathway (Liu et al. 2020). Li and colleagues established a genome-wide correlation between m^6^A and chromatin modification H3K9me2; they found that YTHDC1 directly recruits the KDM3B, a H3K9me2 demethylase, to m^6^A-associated chromatin regions, facilitating H3K9me2 demethylation and transcription (Li et al. 2020). A more recent study showed that *METTL13*-deficient mESCs impair the deposition of multiple heterochromatin marks upon the IAP-type family of endogenous retroviruses. These methylated transcripts are bound by YTHDC1, which has an interaction with METTL3 and in turn contributes to the association of METTL3 with heterochromatin through the physical interplay of METTL3 and H3K9me3 methyltransferase SETDB1 and its cofactor TRIM28 (Xu et al. 2021). Consistently, Liu and colleagues showed that the retrotransposon repression and maintenance of mESCs identity is achieved by the m^6^A reader YTHDC1 (Liu et al. 2021). Mechanistically, YTHDC1 targets these m^6^A-modified retrotransposon transcripts (such as IAPs, ERVK, and LINE1), and that its depletion activates these silenced retrotransposons in a SETDB1-mediated H3K9me3-dependent manner. Furthermore, YTHDC1 and its targets act upstream of SETDB1 to repress the expression of Dux, the major inducer of the two-cell stage (2C)-like state transition, thereby guarding mESCs’ identity (Liu et al. 2021). Collectively, these surprising findings that m^6^A functions in chromatin states may provide a new regulatory mechanism for gene expression in ESC development.

### m^6^A functions in other pluripotent stem cell fate determination

As a conserved mRNA modification, m^6^A has been described as a modulator or balancer, playing critical roles in stem cell fate determination during hematopoiesis, neurogenesis, and gametogenesis.

In the hematopoietic system, m^6^A was demonstrated to promote hematopoietic stem and progenitor cell (HSPC) specification during zebrafish and mouse embryogenesis (Zhang et al. 2017a; Lv et al. 2018) (Fig. 3b). In zebrafish, the lacking of *METTL3* stabilizes the arterial endothelial transcripts, *notch1a* and *rhoca*, with YTHDF2-dependent degradation of m^6^A-modified transcripts, resulting in the continuous activation of the Notch signaling, block of the endothelial-to-hematopoietic transition (EHT), and repression of the earliest HSPC generation (Zhang et al. 2017a). Two key subunits of m^6^A writer complex, *METTL3* and *METTL14*, transcripts are highly expressed in normal HSPCs, suggesting that METTL3 and METTL14 might act as crucial regulators in normal hematopoiesis differentiation (Martin and Park 2018). Removal of METTL3 in mouse cord blood–derived CD34 + HSPCs results in the inhibition of cell proliferation and facilitates myeloid differentiation in an in vitro myeloid differentiation culture condition. Overexpression of wild-type METTL3, instead of the catalytic mutant of METTL3, could inhibit cell differentiation and increase cell growth accordingly (Vu et al. 2017). Consistently, acceleration of myeloid differentiation and the upregulation of differentiation marker genes are also observed upon METTL14 silencing in normal HSPCs. Mechanistically, *METTL14* knockdown downregulates the stability and translation of its targets such as *MYB* and *MYC* in an m^6^A-dependent manner, thereby promoting terminal myeloid differentiation (Weng et al. 2018). These two results suggested that METTL3/14-mediated m^6^A methylation inhibits the differentiation of HSPCs. In contrast, two studies reported that *METTL3*-depleted mice adult hematopoietic system blocks hematopoietic stem cell (HSC) differentiation and accumulates HSCs in adult bone marrow (Lee et al. 2019; Yao et al. 2018). METTL3-mediated m^6^A methylation in *MYC* transcript promotes protein translation in HSCs; depletion of *METTL3* in HSCs downregulates MYC protein level and inhibits HSC differentiation (Lee et al. 2019). Conditional depletion of METTL13 from myeloid cells in mice does not affect myeloid cell number or function (Lee et al. 2019). Additionally, knockout of *YTHDF2* results in functional HSPC expansion (Li et al. 2018b). As such, it needs more researches to reconcile the distinct m^6^A roles in HSPC differentiation.

The abundance of m^6^A in nervous system is higher than other organs, and raises in overall abundance ranged from the embryonic brain to the adult brain, implicating its essential roles in neuro-development. In the embryonic mouse cortex, neural progenitor cells (NPCs) generate neurons locating different cortical layers and sequentially convert to glial production before their depletion during early postnatal stages (Taverna et al. 2014). It is shown that the decreasing METTL3-mediated m^6^A levels leads to the protracted cell-cycle progression of cortical NPCs and reduced differentiation during mouse cortical neurogenesis, in which m^6^A facilitates the decay of neurogenesis-related transcripts (Yoon et al. 2017). Wang and colleagues found that conditional lacking *METTL14* in mouse model displays markedly decreased NPC proliferation and premature differentiation (Fig. 3c). Mechanistically, a genome-wide increase in specific histone modifications corresponds with gene expression alteration and cellular phenotypes, providing an m^6^A-mediated histone modification upon regulating gene expression pathway (Wang et al. 2018). In particular, two specific m^6^A binding proteins in neural cells, FMRP and PRCC2A, are characterized and function in neural differentiation and oligodendrocyte specification, respectively (Edens et al. 2019; Wu et al. 2019). FMRP tunes neural differentiation via promoting nuclear export of methylated transcripts (Edens et al. 2019). PRRC2A controls oligodendrocyte progenitor cell (OPC) proliferation and specification by stabilizing a crucial oligodendroglial lineage determination transcription factor, Olig2, in an m^6^A-dependent manner (Wu et al. 2019).

In the process of gametogenesis, including spermatogenesis and oogenesis, the diploid primordial germ cell undertakes mitosis, meiosis, and cellular differentiation into haploid mature gametes: oocytes in females or spermatozoa in males. This heavily specialized process is accurately regulated at the transcriptional, post-transcriptional, and translational levels. The epitranscriptomic mark m^6^A has shown to play important roles in spermatogenesis and oogenesis. An early clue that m^6^A functions in spermatogenesis was the observation that removal of the m^6^A eraser *ALKBH5* leads to impaired fertility (Zheng et al. 2013). In mammals, spermatogonial stem cells (SSCs) either self-renew or undergo twice mitotic divisions to generate spermatocytes. One study revealed that conditional depletion of either *METTL3* or *METTL14* causes translation dysregulation of m^6^A-modified transcripts, which is required for SSC proliferation and differentiation (Lin et al. 2017). Likewise, another independent study showed that the germ cell-specific *METTL3*-knockout mice impedes spermatogonial specification and blocks meiosis initiation by changing the alternative splicing of spermatogenesis-related genes and the global transcript expression pattern in testes (Xu et al. 2017). As an m^6^A cytoplasmic reader, YTHDC2 is indispensable in cell fate transition during oocyte and spermatogonial development. In spermatogenesis, YTHDC2 interacts with an essential meiosis process factor MEIOC (Abby et al. 2016) and facilitates a clean switch from mitosis to meiosis (Bailey et al. 2017). Furthermore, transcriptome analysis of *YTHDC2*-deficient male germ cells showed that meiosis-related transcripts are downregulated and mitotic cell–related genes are upregulated, ensuring that YTHDC2 recognizes m^6^A-modified germline transcripts to modulate the mitosis closure and transition to meiosis process (Wojtas et al. 2017). YTHDC2 also regulates oogenesis, in which *YTHDC2*-deficient mouse oocytes are arrested in the prophase of meiosis I and cause female fertility (Bailey et al. 2017; Wojtas et al. 2017). However, the specific molecular mechanism underlying what YTHDC2’s m^6^A binding function is and how it regulates oocyte and spermatogonial development needs to be further investigated. Additionally, YTHDC1, as another m^6^A nuclear reader, mediates the polyadenylation process in oocytes by recruitment of 3' end processing factors CPSF6, SRSF3, and SRSF7. Lacking of *YTHDC1* blocks oocyte at the primary follicle stage and fails to achieve transition into secondary follicles (Kasowitz et al. 2018).

### m^5^C functions in stem cell fate

Similar to m^6^A, m^5^C is also a remarkable epitranscriptomic modification and is involved in balancing stem cell self-renewal and specification and maintaining cellular homeostasis of most tissues. The functional studies of either DNMT2 or NSUN2 demonstrate that m^5^C on tRNAs is required for stem cell fate decision. Although DNMT2 is a homolog of DNA methyltransferase, it only has tRNA m^5^C methylation activity and no DNA m^5^C methylation activity (Raddatz et al. 2013). DNMT2 specifically installs m^5^C at the C38 position of tRNAs (Tuorto et al. 2015), and NSUN2 methylates tRNAs at C34, C48, C49, and C50 positions (Blanco et al. 2011; Blanco et al. 2014; Tuorto et al. 2012), which prevent tRNA cleavage. DNMT2-mediated tRNA m^5^C methylation is essential to ensure codon fidelity for accurate protein synthesis, and loss of *DNMT2* disrupts cell-autonomous differentiation during hematopoiesis (Tuorto et al. 2015). NSUN2-mediated m^5^C on tRNAs is required for balancing self-renewal and differentiation, and knockout of *NSUN2* in epidermal stem cells fails to initiate anagen and causes a substantial delay in accurate hair lineage differentiation (Blanco et al. 2011). In male gonads, NSUN2-mediated m^5^C on tRNAs is dispensable in spermatogonial stem cells, and specifically required for the meiotic progression of germ cells into the pachytene stage (Hussain et al. 2013). Likewise, NSUN2 is expressed in early neuroepithelial progenitors during human brain development and required for differentiation capacity. Loss of *NSUN2* increases tRNA fragmentation and impairs migration and differentiation in neuroepithelial stem cells (Flores et al. 2017). A recent finding shows that NSUN2 is also an mRNA m^5^C methyltransferase (Yang et al. 2017a); however, whether the mRNA methylation activity NSUN2 is involved in the aforementioned functions remains unclear. NSUN7 installs m^5^C in eRNA, and loss-of-function mutation of *NSUN7* has been associated with male infertility, implicating that the NSUN7-regulated m^5^C methylation pathway may serve as an essential balancer in germ cell differentiation (Harris et al. 2007). Notably, recent studies discovered that when cells respond to differentiation signals or other cellular and environmental incentives, m^5^C upon tRNA could coordinate translation rates of transcripts encoding functional proteins via the biogenesis of tRFs (Blanco et al. 2016). Removal of NSUN2- and DNMT2-mediated tRNA m^5^C methylation modulates the tRF formation and dysregulated intracellular translation (Tuorto et al. 2015; Blanco et al. 2016). Accordingly, the altered tRNA modification pattern, in particular m^5^C, could shape tRF biogenesis and their intracellular abundances. These generated tRFs influence global and/or gene-specific protein translation by occupying distinctive RNA-binding proteins, and therefore play critical roles in diverse biological developmental processes (Blanco et al. 2016; Zhang et al. 2018; Goodarzi et al. 2015).

## RNA methylation functions in embryonic development

**Fig. 4:**
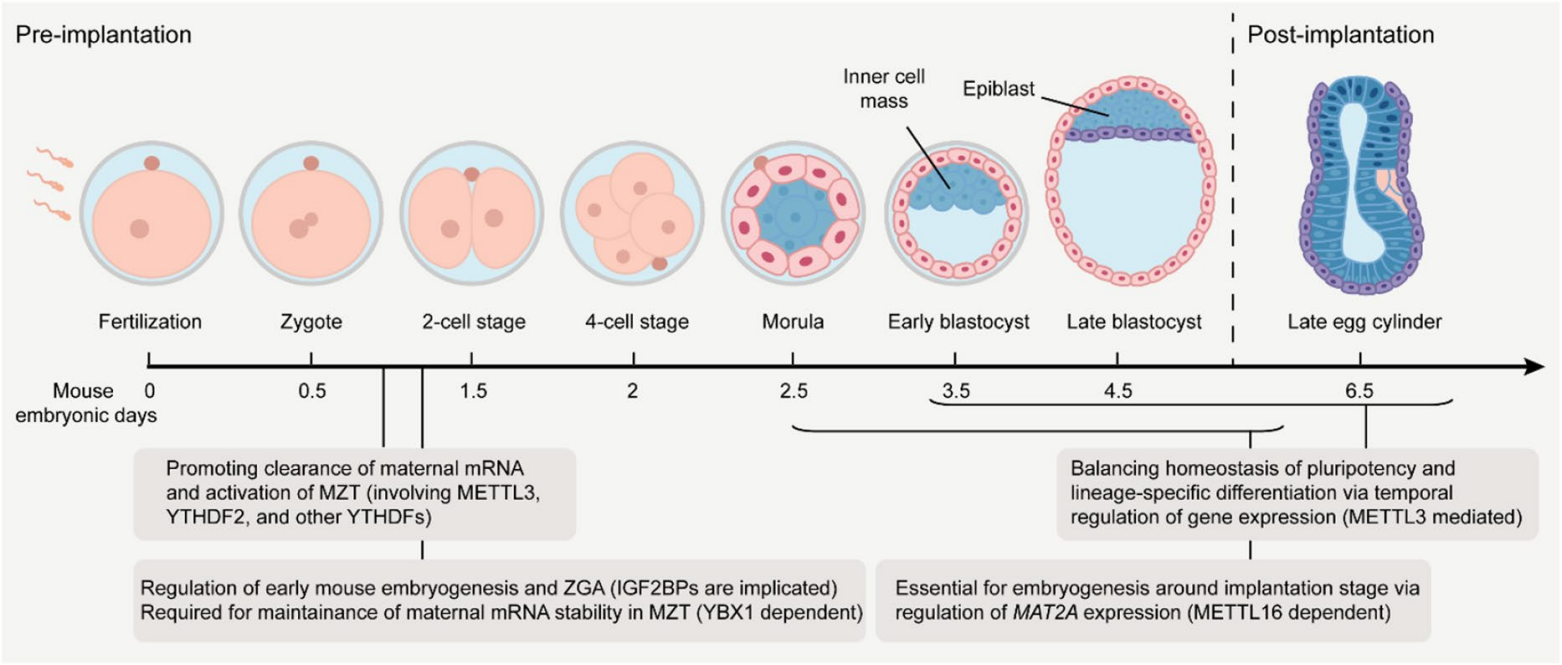
Regulation of RNA methylation in embryonic development. The steps of mouse embryonic development along the timeline are represented, and the key processes implicated by the epitranscriptomic effectors are noted

After gametogenesis, these two terminally specialized gametes are fused into a fertilized egg, followed by a programmed transition into a totipotent and pluripotent embryonic state, and then cell-fate determination and lineage-specific differentiation. In mammalian embryonic development, epigenetic remodeling of histone modifications, DNA methylation, chromatin accessibility, and 3D chromatin organization play indispensable roles in the regulation of gene expression. Additionally, the newly emerging epitranscriptomic marks (m^6^A and m^5^C) are found to function in embryonic development (Fig. 4).

### m^6^A functions in embryo development

Following fertilization, the newly generated zygote sustains a transcriptionally quiescent state and initiates the early maternally programmed embryogenesis because it entirely depends on RNA and proteins from the mature oocyte. However, maternal stores (such as RNA and proteins) are insufficient to support later development, therefore followed by a gradual switch from clearance of maternal stores to zygote genome activation (ZGA). This original program is termed as the maternal-to-zygotic transition (MZT). Recently, increasing studies have illustrated that epitranscriptomic mark m^6^A and its effectors are critical to ensure suitable gene expression in both pre-implantation and post-implantation embryonic development (Zhang et al. 2020). In the pre-implantation embryonic development, m^6^A promotes clearance of maternal mRNA and activation of MZT process. As the essential component of the methyltransferase complex, METTL3-mediated m^6^A installation has a critical role in pre-implantation embryonic development. Knockdown of *METTL3* severely impedes murine oocyte maturation by downregulating mRNA translation efficiency and causes defects in the MZT and ZGA processes (Sui et al. 2020). An mRNA interactome capture study identified m^6^A reader proteins YTHDF1-3 as maternal mRNA-binding partners during zebrafish MZT (Despic et al. 2017), implicating that YTHDF protein–mediated m^6^A regulation may engage in the MZT process. Indeed, over one-third of transcripts in zebrafish are methylated, and the rapid clearance of these maternal mRNAs is accelerated by undertaking an m^6^A-mediated YTHDF2-dependent mRNA decay pathway (Zhao et al. 2017). Lacking of zebrafish *YTHDF2* embryos impedes the degradation of m^6^A-methyalted maternal mRNAs, thereby failing to initiate timely MZT and remaining developmental interruption during larval period (Zhao et al. 2017). Recently, another study reported that m^6^A promotes maternal mRNA deadenylation and degradation, but these effects are not simply dependent on YTHDF2 (Kontur et al. 2020). m^6^A binding proteins YTHDF1-3 function redundantly in ovary development and zebrafish viability, confirming the indispensable of m^6^A in MZT and early embryogenesis (Kontur et al. 2020). Another type of m^6^A readers IGF2BPs can also exert distinctive functions during early embryonic development. Removal of *IGF2BP1* induces cell apoptosis in early mouse parthenogenetic embryogenesis (Hao et al. 2020). Mouse embryos with maternal deletion of *IGF2BP2* are arrested at the 2‐cell‐stage with and ZGA-related genes such as *CCAR1* and *RPS14* are downregulated (Liu et al. 2019). IGF2BP3 is identified as a crucial regulator for maintaining maternal mRNA stability in zebrafish. Lacking of maternal *IGF2BP3* accelerates maternal mRNAs decay prior to MZT and leads to severely impaired development, including abnormal cytoskeleton organization and cell division (Ren et al. 2020). However, whether IGF2BPs serve as m^6^A readers to regulate mRNA stability in ZGA and MZT processes and whether YTHDF2 and IGF2BPs recognize distinct targeted sites in early embryogenesis remain to be deciphered.

In addition to pre-implantation embryogenesis, m^6^A is also indispensable in post-implantation embryonic development. For example, *METTL3*-deficient mouse embryos retain expression of pluripotency factors and display a state of hyperpluripotency, thereby failing to undertake lineage differentiation and leading to lethality during the post-implantation embryonic development (Batista et al. 2014; Geula et al. 2015). Other m^6^A effectors are also crucial in embryonic development. METTL16 is identified as a U6 snRNA m^6^A writer and specifically deposits m^6^A on 3′ UTR hairpin of SAM synthetase *MAT2A* pre-mRNA for intron retention, which promotes MAT2A nuclear degradation and affects SAM homeostasis (Pendleton et al. 2017). Further mechanism study revealed that YTHDC1 recognizes the m^6^A modifications on *MAT2A* hairpin for mRNA degradation (Shima et al. 2017). The function of METTL16 in SAM homeostasis control is required for mouse early embryonic development. *Mettl16*-depleted embryos appear normal before implantation but exhibit developmental arrest around the time of implantation, which is due to the consequence that the SAM limitation leads to massive transcriptome dysregulation at the embryonic day 3.5 blastocysts stage (Mendel et al. 2018). Notably, given that mice with knockout of cytoplasmic m^6^A reader YTHDF2 can survive to late embryonic developmental stages but removal of either *METTL3* or the nuclear m^6^A reader YTHDC1 causes embryonic lethality (Kasowitz et al. 2018; Geula et al. 2015; Batista et al. 2014; Ivanova et al. 2017) implies that m^6^A could play critical roles in the nucleus during embryogenesis.

### m^5^C functions in embryo development

During zebrafish embryogenesis, m^5^C was demonstrated to maintain maternal mRNA stability in MZT process, in which transcripts with more m^5^C-targeted sites and higher m^5^C-methylated levels exhibited more stable expression levels during both 2 to 4-h and 4 to 6-h post-fertilization (hpf) stages, and its binding protein YBX1 functions in impeding early gastrulation arrest. The mechanism study showed that YBX1 directly interacts with m^5^C-containing mRNAs via a π-π interaction and enhances m^5^C-modified maternal genes by recruitment of PABPC1A (Yang et al. 2019). Furthermore, Rai and colleagues indicated that knockdown of *DNMT2* in zebrafish embryos confers deficiency of terminal cellular differentiation toward the retina, liver, and brain. Considering that DNMT2 functions primarily as a genomic DNA methyltransferase and a cytoplasmic tRNA methyltransferase, the results showed that the organ differentiation requires cytoplasmic DNMT2 activity, not in the nucleus, therefore establishing a potential contact between cellular differentiation and tRNA m^5^C modifications (Rai et al. 2007).

## RNA methylation functions in cancer

**Fig. 5:**
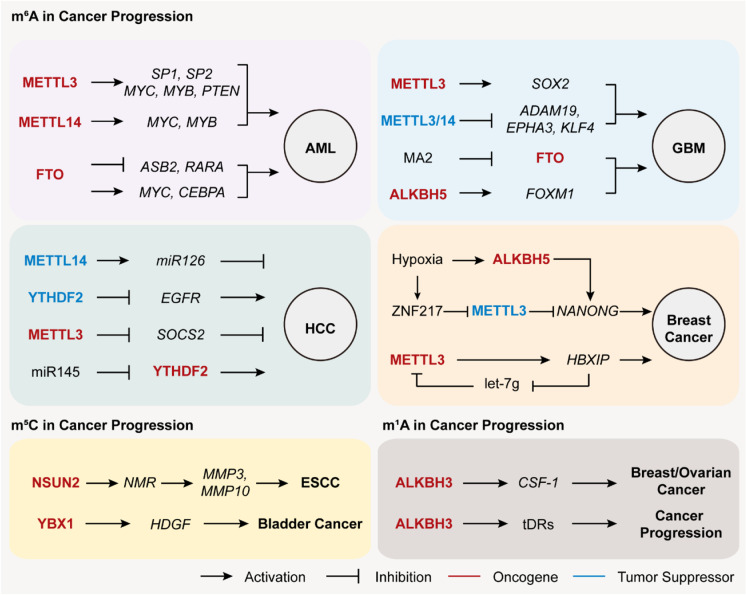
RNA methylation in cancer progression. Epitranscriptomic effectors in diverse types of human cancers play oncogenic (red) or tumor-suppressive roles (blue) in cancer progression. AML, acute myeloid leukemia; GBM, glioblastoma; HCC, hepatocellular carcinoma; ESCC, esophageal squamous cell carcinoma

Cancer stem cells (CSCs) are termed as intratumor heterogeneity and hitherto-unappreciated subclass of neoplastic cells within tumors, and have stem cell-like capacities to self-renew, differentiate, and generate tissue to propagate the tumor (Hanahan and Weinberg 2011). Currently, amount lines of evidence illustrate that the dysregulation of epitranscriptomic modifications is engaged in the pathogenesis of CSCs, including tumorigenesis, cell proliferation, and cell fate decision. Epitranscriptomic modifications are also involved in various cancer subtypes, such as acute myeloid leukemia, glioblastoma, liver cancer, breast cancer, cervical cancer, and bladder cancer (Fig. 5), further implying its potential roles in cancer program.

### m^6^A functions in acute myeloid leukemia

AML is a prevalent hematopoietic malignancy, caused by the advance of stem cell-like renewal capability and the impediment of normal differentiation. Epigenetic modifications, including DNA methylations and histone modifications, are crucial to AML pathogenesis (Chen et al. 2010). Accordingly, many studies have pointed to m^6^A as an important factor for hematopoietic homeostasis and leukemogenesis.

The aberrant m^6^A methylation of mRNA facilitates myeloid differentiation and cell proliferation. The expression levels of METTL3 and METTL14 are more abundant in AML (Vu et al. 2017; Weng et al. 2018), and METTL3 is captured by a genome-wide CRISPR dropout screening, and identified as a necessary gene for AML cell growth (Barbieri et al. 2017). Two independent studies illustrated the oncogenic function of METTL3 in AML. Vu and colleagues found that *METTL3* depletion in AML cells induces cell differentiation and apoptosis, coupled with reduced cell proliferation, by downregulating translation of its RNA targets such as *C-MYC*, *BCL-2*, and *PTEN*, and stimulating the activated AKT level (Vu et al. 2017). Barbieri and colleagues found that METTL3 binds chromatin and localizes to the transcriptional start sites (TSS) of active genes in an independent manner of METTL14 (Barbieri et al. 2017). METTL3 is stably recruited to promoters of transcription factors (SP1 and SP2) by CEBPZ, resulting in m^6^A methylation and enhanced translation. Given that SP1 promotes *MYC* transcription, the resulting increased translation of SP1 in turn leads to upregulated MYC signaling in leukemogenesis (Barbieri et al. 2017). Likewise, METTL14 also functions as an oncogene in the self-renewal of leukemia stem cells (LSCs) and AML maintenance via an m^6^A-meditated MYB/MYC-dependent regulatory pathway (Weng et al. 2018). Additionally, other methyltransferase components such as WTAP and RBM15 are also implicated in leukemogenesis, while its association with m^6^A remains to be elucidated (Bansal et al. 2014; Ma et al. 2001). FTO is significantly upregulated in AMLs with t(11q23)/MLL rearrangements, t(15;17)/PML-RARA, FLT3-ITD, and/or NPM1 mutations, and exerts an oncogenic role in AML by post-transcriptionally downregulating expression of critical transcripts such as *ASB2* and *RARA* through m^6^A demethylation activity (Li et al. 2017b). This regulatory mechanism seems to specifically appear in isocitrate dehydrogenase 1 (IDH1) wild-type AML cells, while IDH mutants induce the conversion of α-KG to R enantiomer of 2-hydroxyglutarate (R-2HG). The demethylation activity of FTO is suppressed by R-2HG and plays critical oncogenic roles in leukemia through an FTO/m^6^A/MYC-CEBPA axis (Su et al. 2018).

### m^6^A functions in glioblastoma

Glioblastoma (GBM) is one of the most prevailing primary malignant brain tumors, with a median survival of around 10 months (Rick et al. 2018). GBM is characterized by notable heterogeneity between intra- and inter-tumor and contains apical cells of cellular hierarchies with stem-like properties. Glioblastoma stem cells (GSCs) belong to a subclass of CSCs with capacities to facilitate self-renewal, tumor growth, and invasion (Kalkan 2015). Recent studies uncovered that m^6^A is correlated with GSC self-renewal, tumorigenesis, and therapy resistance in GBM.

Recently, although there are notable exceptions, several studies indicate that m^6^A generally impairs self-renewal and tumorigenesis in GBM. m^6^A methylation levels are significantly elevated upon induced GSC differentiation. Knockdown of either *METTL3* or *METTL14* enhances GSC growth and self-renewal and upregulates expression level of oncogenic genes with downregulated m^6^A methylation, including *ADAM19*, *EPHA3*, and *KLF4* (Cui et al. 2017). By contrast, it is reported that *METTL3* expression level is upregulated in GSCs and attenuated upon differentiation. *METTL3*-silenced CSCs inhibit GSC maintenance and enhance radiation sensitivity by stabilizing the downstream target *SOX2* with recruitment of the HuR, suggesting that METTL3 functions as an oncogene in GBM (Visvanathan et al. 2018). These conflicting results are possibly caused by the variability of GBM cell types. Additionally, ALKBH5 is highly expressed in GSCs and positively functions in cell proliferation, self-renewal, and tumorigenicity. Removal of *ALKBH5* increases m^6^A methylation on *FOXM1* pre-mRNA and downregulates *FOXM1* transcript expression level, leading to impaired proliferation and tumorigenesis of GSCs. The ALKBH5-FOXM1 regulatory pathway can be further strengthened by a lncRNA antisense to FOXM1 (FOXM1-AS) (Zhang et al. 2017b). Likewise, FTO undertakes oncogenic functions in GBM program (Cui et al. 2017; Su et al. 2018), and MA2, the FTO chemical inhibitor, can coordinately prolong the life span in GSC-transplanted mice (Cui et al. 2017), suggesting that FTO-regulated m^6^A awaits to be a promising therapeutic target for GBM treatment.

### m^6^A functions in hepatocellular carcinoma

Hepatocellular carcinoma (HCC) is one of the most commonly diagnosed primary liver cancer and rates as the fifth malignant tumor worldwide. Aberrant expression of m^6^A effectors is associated with HCC progression. For example, METTL3 negatively predicts prognostic survival, and its overexpression remarkably enhances HCC growth. Knockdown of *METTL3* significantly reduces HCC tumorigenicity by increasing *SOCS2* expression via an m^6^A-mediated YTHDF2-dependent mRNA decay pathway (Chen et al. 2018). However, Ma and colleagues showed that METTL14 exhibits a converse role in HCC development. Decreased METTL14 is observed in HCC and closely associated with tumor metastasis and prognosis. In particular, METTL14 interacts with DGCR8 and positively processes pri-miR126 into mature miR126, a typical tumor repressor in HCC metastasis, in an m^6^A-dependent manner (Ma et al. 2017). Moreover, two studies exhibited controversial functions of YTHDF2 in the HCC program. One study showed that miR-145 is suppressed in HCC patients and downregulates the expression level of oncogenic gene, *YTHDF2* (Yang et al. 2017b). Zhong and colleagues found that YTHDF2 represses cell proliferation and growth in HCC and functions as a HCC tumor suppressor via destabilizing *EGFR* transcript (Zhong et al. 2019).

### m^6^A functions in breast cancer

Breast cancer is the highest incidence of malignant tumor among women. Aberrant expression of m^6^A effectors are involved in CSC maintenance under tumor microenvironment in breast cancer. As the typical feature of tumor microenvironment, hypoxia enhances ALKBH5 expression in breast cancer stem cells (BCSCs) in a HIF-dependent manner. The upregulated ALKBH5 decreases m^6^A methylation in *NANOG* transcript for mRNA stabilization, thereby enhancing BCSC percentage and phenotype (Zhang et al. 2016a). Meanwhile, zinc finger protein 217 (ZNF217) expression is also upregulated in an HIF-dependent manner under hypoxia. The increased ZNF217 leads to m^6^A hypomethylation by disrupting METTL3-mediated m^6^A methylation and cooperating with ALKBH5, therefore resulting in *NANOG* upregulation and promoting BCSC maintenance (Zhang et al. 2016b). Another study showed that METTL3 is highly expressed in clinical breast cancer samples and engages in a positive feedback loop, cooperated with HBXIP and let-7g miRNA (HBXIP/let-7g/METTL3/HBXIP), to accelerate proliferation in breast cancer. Mechanistically, the oncoprotein HBXIP upregulates *METTL3* by suppressing miRNA let-7g, and the increased METTL3, in turn, enhances *HBXIP* expression through the promotion of m^6^A modification (Cai et al. 2018).

### m^5^C functions in cancer

NSUN2 has been identified as an MYC target correlated to the MYC-dependent proliferation in tumors (Frye and Watt 2006), and DNMT2 is highly expressed in various tumor tissue cells (Forbes et al. 2015), implying that DNMT2 functions an oncogenic role in tumorigenesis. Usage of azacytidine could inhibit DNMT2-mediated RNA methylation and reduce metabolism of cancer cells (Schaefer et al. 2009). A homozygous splice mutation in NSUN2 brings about a loss of m^5^C on known NSUN2 targets in tRNA, and likely acts as a cause of a Dubowitz-like syndrome (Martinez et al. 2012). These potential lines of evidence demonstrate that tRNA m^5^C and its effectors may function in cancer development.

Recently, two independent studies revealed that m^5^C methylation in mRNA and lncRNA enhances the cancer susceptibility. One study showed that NSUN2 enhances tumor metastasis by m^5^C methylation of *NMR* lncRNA in esophageal squamous cell carcinoma (ESCC). The m^5^C methylated *NMR* along with BPTF play oncogenic roles in promoting expression of key drivers of esophageal cancer such as *MMP3* and *MMP10* (Li et al. 2018a). The other research showed that NSUN2-mediated m^5^C methylation highly occurred on oncogenic mRNAs of human urothelial carcinoma of the bladder (UCB). Among these, the heparin-binding growth factor (*HDGF*) transcript is methylated by NSUN2 and stabilized by m^5^C reader YBX1, which in turn facilitates the pathogenesis of bladder cancer (Chen et al. 2019a). Together, m^5^C on tRNA may function in modulating cancer metabolism, and m^5^C on mRNA potentially plays roles in enhancing the stability of cancer-related transcripts. Nevertheless, the complete prospect of functions of m^5^C in cancers awaits to be discovered.

### m^1^A functions in cancer

Compared with normal tissues, ALKBH3 is highly expressed in pancreatic (Konishi et al. 2005), lung (Tasaki et al. 2011), and urothelial cancers (Shimada et al. 2012), and is evenly identified as the high-grade prostate cancer marker, implying that ALKBH3 may function in the tumorigenesis process and would be helpful for the early diagnosis.

In diverse cancer subtypes, ALKBH3 functions as an m^1^A demethylase and plays an oncogenic role in cancer progression via various regulatory mechanisms. In breast and ovarian cancer, ALKBH3 functions as an oncogene for cancer invasiveness via stabilizing colony-stimulating factor 1 (*CSF-1*) transcript in an ALKBH3-mediated m^1^A demethylation manner (Woo and Chambers 2019). As a tRNA m^1^A demethylase, the ALKBH3-mediated demethylation responds sensitively to angiogenin (ANG)-dependent tRNA cleavage, followed by generating tRNA-derived sRNAs (tDRs). The produced tDRs are engaged in ALKBH3-mediated cancer progression via modulating ribosome assembly and preventing apoptosis pathway triggered by cytochrome *c* (Cyt *c*) (Chen et al. 2019b). Additionally, ALKBH3 is also responsible for DNA damage caused by alkylating agents (Duncan et al. 2002; Dango et al. 2011), which means that ALKBH3 may play potential roles in tumorigenesis by an RNA m^1^A-independent manner.

## Conclusions

Recently, increasing studies showed that epitranscriptomic marks are established as critical regulators in gene expression, and are of underlying importance for diverse biological developments. In the process of stem cell differentiation, the dynamic deposition of RNA modifications maintains the balance between pluripotency and differentiation into downstream lineages in an ordered manner to enable appropriate cellular development. As for early embryogenesis, RNA modifications, in particular m^6^A, are critical to eliminate maternal mRNA in an m^6^A-mediated YTHDF2-dependent mRNA decay pathway and to promote the ZGA process, ensuring suitable gene expression. Furthermore, RNA modifications along with their effectors also have oncogenic or tumor-suppressor functions in the cancer pathogenesis, including tumorigenesis, cancer cell proliferation, and cancer cell fate decision of numerous cancer types. In some cases, the same enzyme can play conflicting functions in diverse cancer types, which can be explained by tumor specificity as well as the diversity of RNA modifications. Peculiarly, both m^6^A writers (METTL3 and METTL14) and m^6^A eraser (FTO) function as an oncogene in AML development and progression. Considering that there are numerous m^6^A-modified tumor-related mRNAs and opposite roles of m^6^A binding proteins (YTHDF2 and IGF2BPs), the dysregulation of m^6^A installation could disrupt the homeostasis of post-transcriptional gene expression, thereby leading to oncogenic function in cancerogenesis. Notably, increasing studies showed that METTL3-mediated m^6^A methylation modulates chromatin state in mESCs, providing a new regulatory mechanism in gene expression of development.

## Data Availability

Not applicable.
